# *ITGB3BP* is a potential biomarker associated with poor prognosis of glioma

**DOI:** 10.1111/jcmm.17127

**Published:** 2021-12-24

**Authors:** Zhendong Liu, Binfeng Liu, Lu Bian, Hongbo Wang, Yulong Jia, Yubo Wang, Wang Zhang, Yanbiao Wang, Zhibin Han, Xingbo Cheng, Xiaoyu Lian, Zhishuai Ren, Yanzheng Gao

**Affiliations:** ^1^ Department of Surgery of Spine and Spinal Cord Henan Provincial People's Hospital Henan Province Intelligent Orthopedic Technology Innovation and Transformation International Joint Laboratory Henan Key Laboratory for Intelligent Precision Orthopedics People's Hospital of Zhengzhou University People’s Hospital of Henan University Henan China; ^2^ Zhengzhou University People's Hospital Henan Provincial People's Hospital Henan China; ^3^ Department of Dermatology Henan University People's Hospital Henan Provincial People's Hospital Henan China; ^4^ Department of Neurosurgery of the Henan Provincial People's Hospital Henan China; ^5^ College of Agriculture Henan University of Science and Technology Luoyang China; ^6^ Department of Neurosurgery of the First affiliate Hospital of Harbin Medical University Harbin China

**Keywords:** biomarker, clinical features, glioma, *ITGB3BP*, prognosis

## Abstract

Despite the growing recognition of *ITGB3BP* as an essential feature of various cancers, the relationship between *ITGB3BP* and glioma remains unclear. The main aim of this study was to determine the prognostic and diagnostic value of *ITGB3BP* in glioma. RNA‐Seq and microarray data from 2222 glioma patients were included, and we found that the expression level of *ITGB3BP* in glioma tissues was significantly higher than that in normal brain tissues. Moreover, *ITGB3BP* can be considered an independent risk factor for poor prognosis and has great predictive value for the prognosis of glioma. Gene Set Enrichment Analysis results showed that *ITGB3BP* contributes to the poor prognosis of glioma by activating tumour‐related signalling pathways. Some small‐molecule drugs were identified, such as hexestrol, which may specifically inhibit *ITGB3BP* and be useful in the treatment of glioma. The TIMER database analysis results revealed a correlation between the expression of *ITGB3BP* and the infiltration of various immune cells in glioma. Our findings provide the first evidence that the up‐regulation of *ITGB3BP* correlates with poor prognosis in human glioma. Thus, *ITGB3BP* is a potential new biomarker that can be used for the clinical diagnosis and treatment of glioma.

## 1 INTRODUCTION

Glioma is the most common malignant disease of the central nervous system, originating from neural stem cells with significant heterogeneity.^1^ Due to the malignant characteristics of glioma, such as infiltration and rapid growth, glioma patients often have a higher disability and mortality rate. Therefore, glioma poses a severe threat to human health.^2^ Presently, surgery combined with radiotherapy and chemotherapy is the primary treatment for reducing the disability rate and extending the survival time of glioma patients.^3^ However, because the tumour tissue boundary is unclear, the surgical resection rate is low, and the recurrence rate is high, leading to poor overall treatment outcomes. In recent years, studies on immune checkpoints have received the most attention, but the long‐term survival rate and cure rate of glioma still have not been effectively improved.^4^, ^5^, ^6^ This is mainly due to the specific pathogenesis of glioma, which remains unknown. Therefore, it is of great clinical significance to gain insight into the pathogenesis of glioma, optimize the treatment plan and identify potential clinical treatment targets.

Recently, many biomarkers have been associated with glioma diagnosis, treatment and prognosis. O6‐methylguanine‐DNA methyltransferase (MGMT) is a DNA damage repair protein. Strict regulation of MGMT methylation can reduce MGMT expression levels and promote cell apoptosis, improving responsiveness to temozolomide (TMZ) and moderately improving the survival rate.^7^, ^8^, ^9^ Additionally, the co‐deletion of 1p/19q and IDH1/2 mutations in patients with low‐grade glioma indicate a better clinical prognosis; however, the effect of these molecular events in glioblastoma (GBM) is unclear.^10^, ^11^ Moreover, IDH mutations usually occur in low‐grade glioma (LGG) and secondary glioblastoma, which may be related to the effect of d‐2hg on DNA demethylase, and often occur simultaneously with a p53 mutation or 1p/19q co‐deletion.^12^, ^13^ In addition, ATP‐binding cassette (ABC) transporters, nestin, PI3K and CD133 have also been reported as glioma biomarkers.^14^, ^15^, ^16^, ^17^ Although many biomarkers have been identified, because of the variety and large number of glioma clinical subtypes, there are no specific biomarkers with diagnostic and prognostic value for each subtype. Therefore, there is an urgent need to identify specific biomarkers.

Invasion and metastasis are the hallmarks of malignant tumours, and the tumour microenvironment (TME) is the main factor mediating tumour metastasis.^18^ Integrin‐binding proteins play a pivotal role in the TME and regulate the occurrence and development of tumours through various signalling pathways. Studies have shown that the integrin subunit β3‐binding protein (*ITGB3BP*) is significantly up‐regulated via the TGF‐β pathway, promoting the metastasis and invasion of breast cancer cells under hypoxic conditions, and is also an important element leading to poor breast cancer prognosis.^19^, ^20^ Moreover, the co‐expression of *ITGB3BP* and cyclin‐dependent kinase inhibitor 2A *(CDKN2A)* is very common in human solid tumours, particularly in bladder cancer.^21^ In addition, *ITGB3BP*, an anti‐apoptotic gene, is involved in cell adhesion, and its expression is up‐regulated in colorectal cancer.^22^ From the evidence above, it is clear that *ITGB3BP*, as a pathogenic gene, plays an important regulatory role in the pathological process of a variety of tumours. However, to date, there are no studies on the relationship between *ITGB3BP* and glioma or the biological function of *ITGB3BP* in glioma.

Glioma research is one of the leading research directions for neuroscience surgeons. Therefore, we collected thousands of tissue samples from multiple databases for comprehensive analysis to reveal the regulatory effect of *ITGB3BP* in the pathological process of glioma. In this study, we investigated the biological function of *ITGB3BP* in glioma and its relationship with survival prognosis for the first time. Our findings provide the first evidence that *ITGB3BP* is a glioma oncogene that can affect the survival time of patients through the cell cycle and other signalling pathways; thus, targeting *ITGB3BP* may be a promising treatment strategy.

## 2 MATERIALS AND METHODS

### 2.1 Data collection

The Chinese Glioma Genome Atlas (CGGA; http://www.cgga.org.cn/ ) database is used for data storage and analysis and includes genomic and detailed clinical data of thousands of glioma patients. We compiled data from 748 patients in the CGGA RNA‐Seq database and 268 patients in the CGGA microarray database. Tables S1 and S2 present the detailed clinical data.

The Cancer Genome Atlas (TCGA, http://cancergenome.nih.gov/) database collects various cancer data, allowing researchers to forge a deeper understanding of cancer and establish a foundation for accurate diagnosis and treatment.^23^ We compiled data from 653 glioma patients from TCGA RNA‐Seq database. Table S3 presents the specific clinical information.

Gene Expression Omnibus (GEO, https://www.ncbi.nlm.nih.gov/geo/) includes high‐throughput gene expression data referred to by worldwide research establishments and is the largest and most comprehensive public gene expression data resource.^24^ We retrieved seven glioma datasets from GEO (GSE4290, GSE50161, GSE4412, GSE43378, GSE50025, GSE74187 and GSE83300). GSE4290 contained data from 77 glioma tissues and 23 normal brain tissues, GSE50161 contained data from 34 glioma tissues and 13 normal brain tissues, and the remaining five datasets contained data from 279 glioma samples. These datasets were used to verify the difference in *ITGB3BP* expression between glioma and normal tissues and the relationship between the expression level of *ITGB3BP* and the survival prognosis of patients with glioma.^25^

Data on the expression of *ITGB3BP* in various human tumour tissues were obtained from the Gene Expression Profiling Interactive Analysis (GEPIA, http://gepia.cancer‐pku.cn/.) database, which is a public database recently developed by a Chinese research group to analyse gene expression profiles of cancer and normal tissues, bridging the gap in cancer genomics data and supporting all expression analysis data at the isomer level.^26^ The GEPIA data in this study included that from 163 high‐grade glioma (HGG) samples and 207 normal brain samples.

The Human Protein Atlas (HPA; http://www.proteinatlas.org/ ) database provides a complete map of protein expression in all major tissues and organs of the human body and has the highest level of specificity, versatility and reproducibility among related databases.^27^ We examined the protein expression of *ITGB3BP* in normal brain tissue and in HGG and LGG to understand the variation in protein expression in the collected tumour samples.

### 2.2 Gene set enrichment analysis

Gene Set Enrichment Analysis (GSEA) is a tool for analysing microarray data at the whole‐genome level. GSEA identifies differentially expressed genes among whole‐genome datasets, allowing researchers to interpret the microarray results more efficiently and accurately.^28^, ^29^ We classified the data from the CGGA RNA‐Seq, CGGA microarray and TCGA RNA‐Seq digital datasets into groups with high and low *ITGB3BP* expression levels. GSEA 4.0.jar was used to investigate the signalling pathways related to *ITGB3BP*. The number of permutations was set at 1000, and ‘KEGG cell signalling pathways’ was selected for the gene data library.

### 2.3 Target drug explanation

The connectivity map (CMap) is a unique pharmacogenomic database devoted to revealing the functional relationships among drugs, genes and diseases through changes in gene expression. Using the GPL570 platform, we transformed genes (10 positively correlated and 10 negatively correlated) related to *ITGB3BP* into gene probe information via co‐expression analysis. Drugs corresponding to the gene probe files were searched using https://portals.broadinstitute.org/cmap/. *p* < 0.01 and enrichment <−0.75 were set as the thresholds to identify inhibitors of *ITGB3BP*.

### 2.4 Meta‐analysis

We conducted a systematic search from databases such as PubMed, Web of Science and Embase and found no prior research report on the relationship between *ITGB3BP* and the prognosis of glioma. Since this study explored the prognostic role of *ITGB3BP* in glioma for the first time, we could not obtain relevant data from published articles. Therefore, we used meta‐analysis to reveal the overall prognostic significance of *ITGB3BP* in glioma patients from the CGGA RNA‐Seq, CGGA microarray, TCGA RNA‐Seq and GEO datasets. The hazard ratio (HR) and 95% confidence interval (CI) were used to evaluate the correlation between the expression of *ITGB3BP* and the survival prognosis of patients with glioma. In addition, the heterogeneity between different data sets was evaluated using the *Q* test (*I* ^2^ statistics). When *I* ^2^ was <50%, the fixed‐effects model was used for meta‐analysis; otherwise, the random‐effects model was implemented.

### 2.5 Analysis of immune infiltration

Tumour Immune Estimation Resource (TIMER, https://cistrome.shinyapps.io/timer/), based on RNA‐Seq expression profile data, is widely used to detect the infiltration of immune cells in tumour tissues.^30^ The database uses high‐throughput sequencing data to analyse the infiltration of immune cells (CD8^+^ T cells, B cells, neutrophils, CD4^+^ T cells, dendritic cells and macrophages) in tumour tissues. We used the TIMER ‘gene’ module to evaluate the relationship between the level of *ITGB3BP* expression and the level of immune cell infiltration of glioma. We also analysed the relationship between *ITGB3BP* and immune escape genes (*CD274*, *PDCD1* and *PDCD1LG2*) to explore whether *ITGB3BP* affects the prognosis of glioma patients by mediating immune escape.

### 2.6 Cell culture and tissue preparation

The human glioma cell lines (T98, U251 and A172) and the corresponding normal cell line (HA) were provided by Procell Life Science & Technology Co., Ltd. Cells were cultured in DMEM with 10% FBS (Gibco), 100 U/ml penicillin and 100 mg/ml streptomycin (Invitrogen) at 37°C with 5% CO_2_. The cells were passaged when they reached about 80%–90% confluence and were detached with 0.25% trypsin (containing 0.01% ethylenediaminetetraacetic acid [EDTA] at a ratio of 1:3). Seventeen glioma tissues and ten normal brain tissues were obtained from the Henan Provincial People's Hospital (Zhengzhou, China) during surgery and stored at −80°C prior to analysis. The study protocol was approved by the Ethics Committee of Henan Provincial People's Hospital (Zhengzhou, China).

### 2.7 RT‐qPCR

RT‐qPCR was used to detect the expression of *ITGB3BP* in glioma cells and tissues. Total RNA Kit I (Omega, Biotek) and Trizol Reagent (Thermo Fisher Scientific) were used to extract total RNA from cells and tissues respectively. RNA reverse transcription was performed using NovoScript Plus All‐in‐one 1st Strand cDNA Synthesis SuperMix (Novoprotein). NovoStart SYBR qPCR SuperMix Plus (Novoprotein) was used to perform RT‐qPCR to determine RNA expression levels. GAPDH expression was used to normalize *ITGB3BP* expression, and the primer sequences of *ITGB3BP* and *GAPDH* were as follows: *ITGB3BP*‐Forward: GCGTTTCCTTTGGCGGATTT, *ITGB3BP*‐Reverse: AGTGATCTTTTAACAGGCATTCTGA, GAPDH‐Forward: CAAGGTCATCCATGACAACTTTG, and GAPDH‐Reverse: GTCCACCACCCTGTTGCTGTAG.

### 2.8 Statistical analysis

Perl and R (v4.0.5) were used for all data analyses in this study. The limma package was used to analyse the *ITGB3BP* expression levels in glioma and normal brain tissues, and the Kaplan‐Meier method was used to identify the association between *ITGB3BP* levels and OS in glioma patients. Univariate and multivariate analyses were performed using Cox or multi‐Cox regression, respectively, to determine whether *ITGB3BP* could be used as an independent predictor of glioma prognosis. Cox regression was used for the receiver operating characteristic (ROC) and area under the curve (AUC) analyses. The Wilcoxon or Kruskal‐Wallis tests were used to investigate the link between the clinical characteristics or prognosis of tumour patients and the aberrant expression of *ITGB3BP*. The co‐expression of *ITGB3BP* with other genes was evaluated using the cor.test function in R. Statistical significance was set at *p* < 0.05.

## 3 RESULTS

### 3.1 Aberrant overexpression levels of *ITGB3BP* were observed in glioma

**FIGURE 1:**
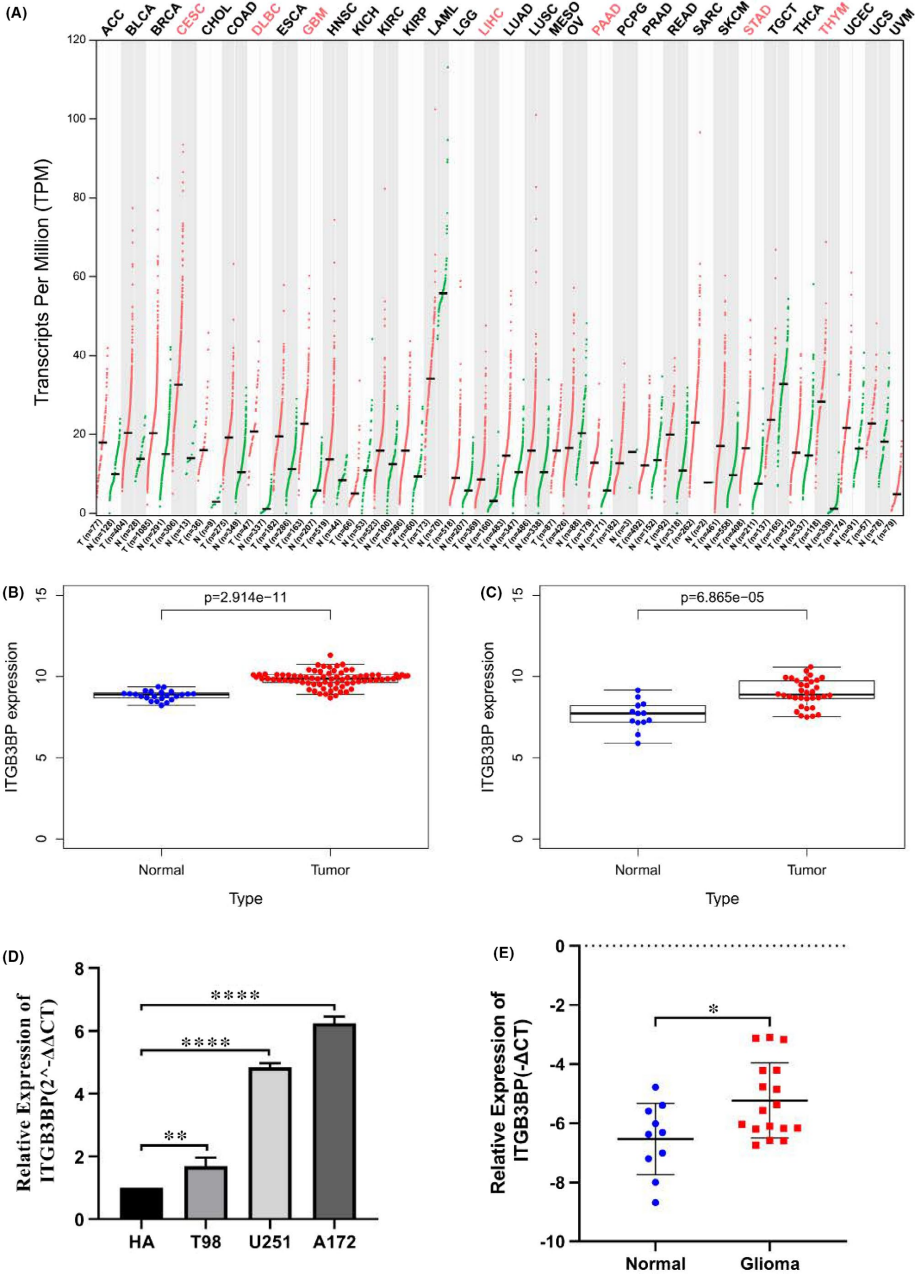
*ITGB3BP* was overexpressed in glioma. A: The expression level of *ITGB3BP* in different varieties of tumours. Different colours represent different tumours. Red indicates tumours with an increased expression level of *ITGB3BP*. Black indicates tumours exhibiting no relationship with the expression level of *ITGB3BP*. B: GSE4290: the expression level of *ITGB3BP* in normal brain tissues and glioma tissues. C: GSE50161: the expression level of *ITGB3BP* in normal brain tissues and glioma tissues. D: The expression level of *ITGB3BP* in T98, A172 and U251 cell lines was higher than that in the HA cell line. E: *ITGB3BP* was up‐regulated in glioma tissues compared with that in normal tissues. *p < 0.05, **p < 0.01, ***p < 0.001, ****p < 0.0001. *ITGB3BP*, integrin subunit β3‐binding protein

To determine the expression level of *ITGB3BP* in glioma, we first analysed the mRNA expression of *ITGB3BP* in the GEPIA database (Figure 1A). The results showed that the expression level of *ITGB3BP* in GBM was significantly increased (GBM: 163; normal brain tissue: 207). To compile credible results, we further used the data in the GEO dataset to compare the expression levels of *ITGB3BP* in glioma tissues and normal tissues. This finding was unexpected and suggested that the average expression level of *ITGB3BP* in glioma tissue was higher than that in normal brain tissue (Figure 1B,C). We also analysed the expression level of *ITGB3BP* protein in glioma using immunohistochemistry (IHC) data. The protein level was not significantly increased in glioma samples compared to that in normal brain tissue samples (Figure S1). Finally, to further validate the changes in the mRNA expression level of *ITGB3BP* in glioma, we used RT‐qPCR to verify the expression of *ITGB3BP* in glioma cell lines (T98, U251 and A172) and tissues. As predicted, the mRNA expression level of *ITGB3BP* in glioma cell lines and tissues was significantly up‐regulated compared to that in the corresponding normal cell lines (HA) and normal tissues (Figure 1D,E). In general, these observations indicate that the mRNA expression level of *ITGB3BP* in glioma tissues is significantly up‐regulated, suggesting that *ITGB3BP* may act as a tumour promoter and play a significant role in tumourigenesis.

### 3.2 Up‐regulated *ITGB3BP* and clinical characteristics were significantly related to glioma prognosis

**FIGURE 2:**
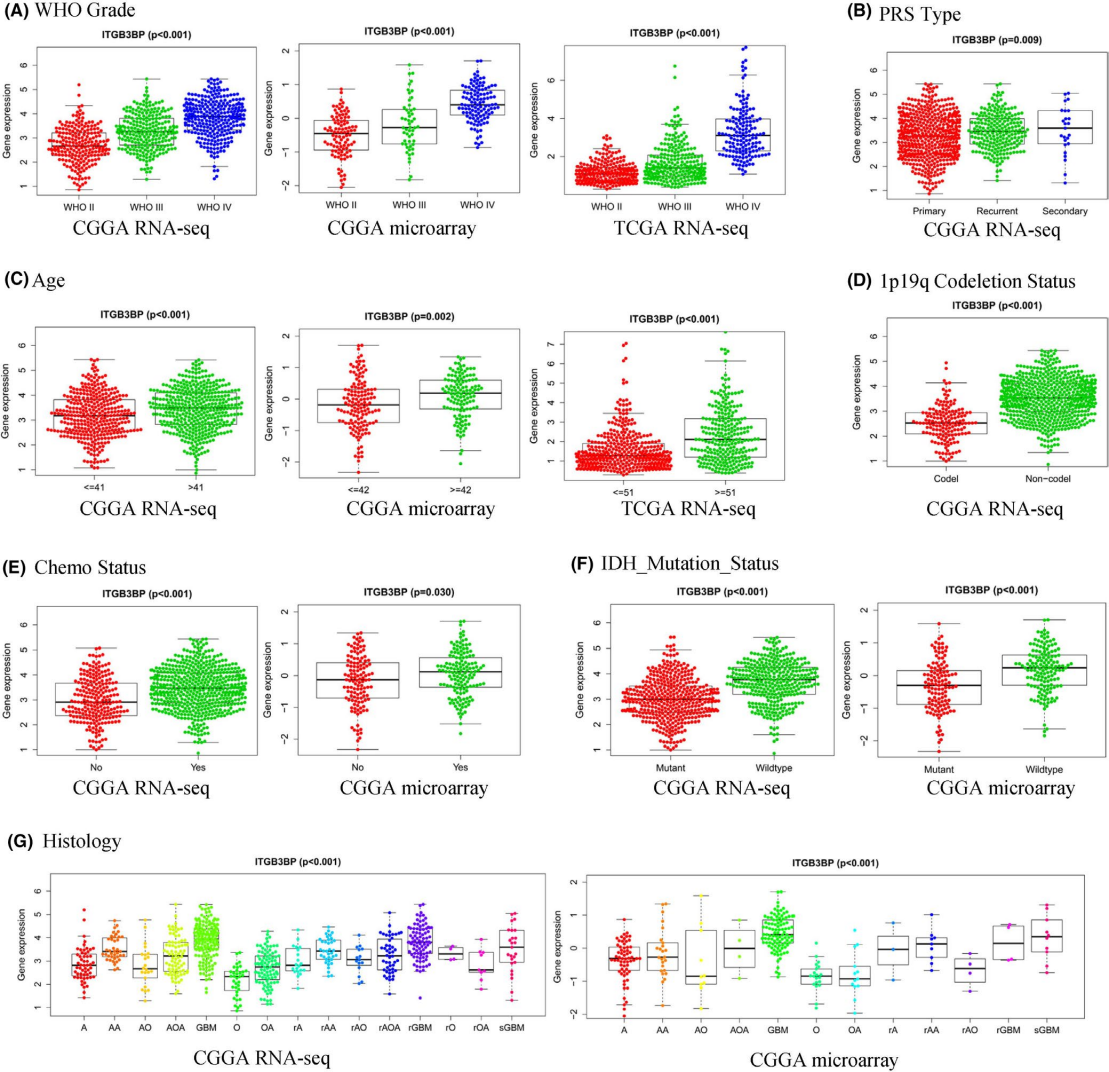
Relationship between *ITGB3BP* expression and clinical features associated with glioma prognosis. A: World Health Organization Grade. B: PRS Type. C: Age. D: 1p19q Codeletion Status. E: Chemo Status. F: IDH Mutation Status. G: Histology. *ITGB3BP*, integrin subunit β3‐binding protein; IDH, isocitrate dehydrogenase

Due to the abnormally high expression of *ITGB3BP* in glioma tissues, we then explored whether there is a relationship between the expression level of *ITGB3BP* and clinical features related to the prognosis of glioma. For example, PRS type, age, chemo status, WHO grade and history are associated with poor prognosis, while the 1p19q co‐deletion and IDH mutations are associated with better prognosis. According to the Wilcoxon and Kruskal‐Wallis analysis results (Figure 2A), WHO grade was positively correlated with *ITGB3BP* expression, the higher the grade, the higher the gene expression level. The expression level of *ITGB3BP* in secondary and recurrent glioma was significantly higher than that in primary glioma (Figure 2B). *ITGB3BP* was also positively correlated with age and chemotherapy (Figure 2C,E), and the expression level of *ITGB3BP* in grade IV glioma (glioblastoma) was significantly higher than that in grade III glioma (anaplastic astrocytoma, anaplastic oligodendroglioma and anaplastic oligoastrocytoma), which in turn showed higher *ITGB3BP* expression levels than grade II glioma (astrocytoma, oligodendroglioma and oligoastrocytoma) (Figure 2G). However, the expression level of *ITGB3BP* in the 1p19q co‐deletion and IDH mutation group was significantly lower than that in the wild‐type group (Figure 2D,F). These results suggest that *ITGB3BP* is associated with poor prognosis in patients with glioma.

### 3.3 Poor prognosis of glioma was associated with abnormal overexpression of *ITGB3BP*

**FIGURE 3:**
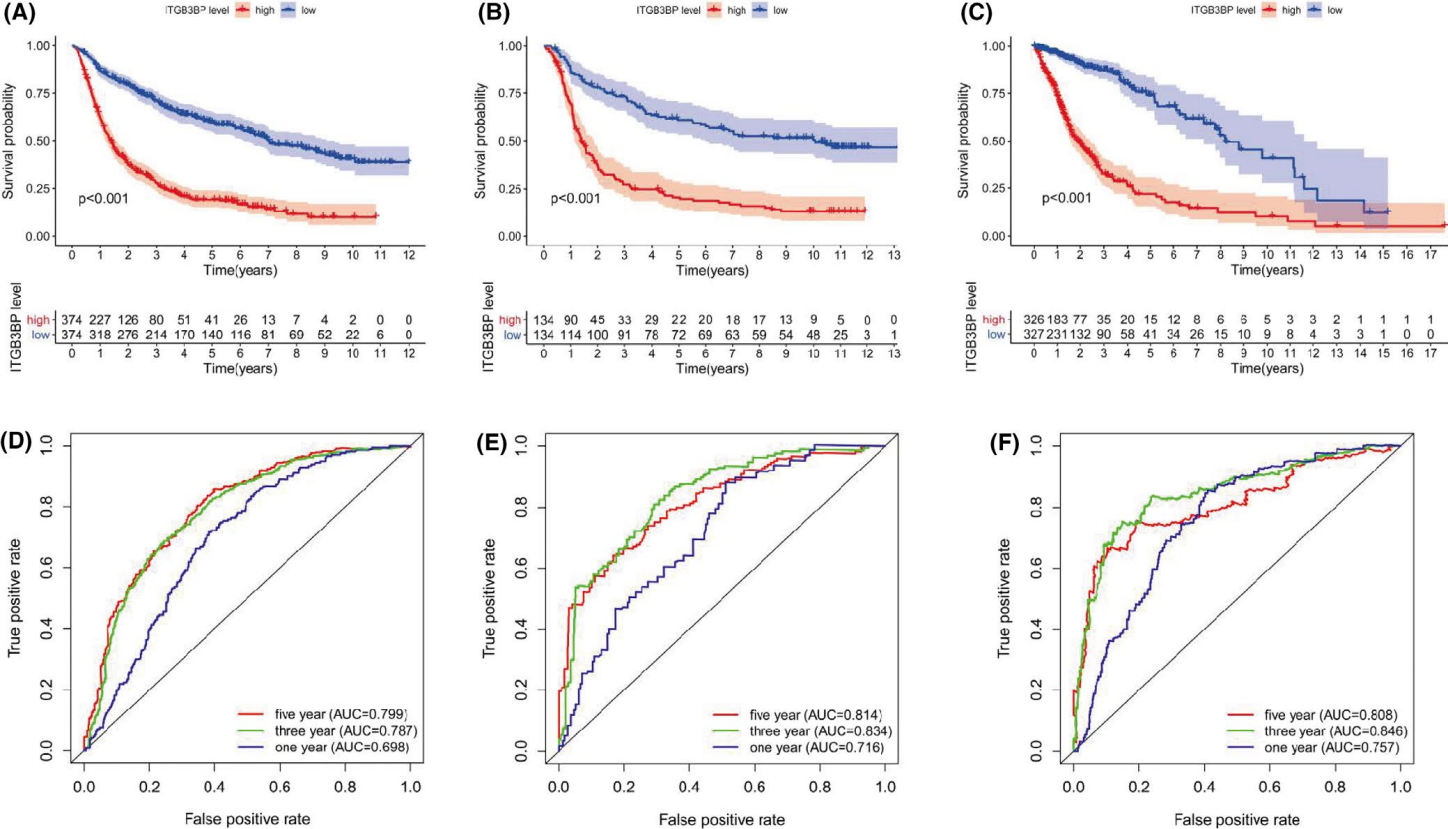
*ITGB3BP* exhibited prognostic value for predicting the survival of patients with glioma. The higher the expression of *ITGB3BP*, the shorter the survival period. A–C: The effect of the expression level of *ITGB3BP* on overall survival based on the CGGA RNA‐Seq, CGGA microarray and TCGA RNA‐Seq datasets. D–F: ROC curves based on CGGA RNA‐Seq, CGGA microarray and TCGA RNA‐Seq datasets. CGGA, Chinese Glioma Genome Atlas; *ITGB3BP*, integrin subunit β3‐binding protein; ROC, receiver operating characteristic; TCGA, The Cancer Genome Atlas

**FIGURE 4:**
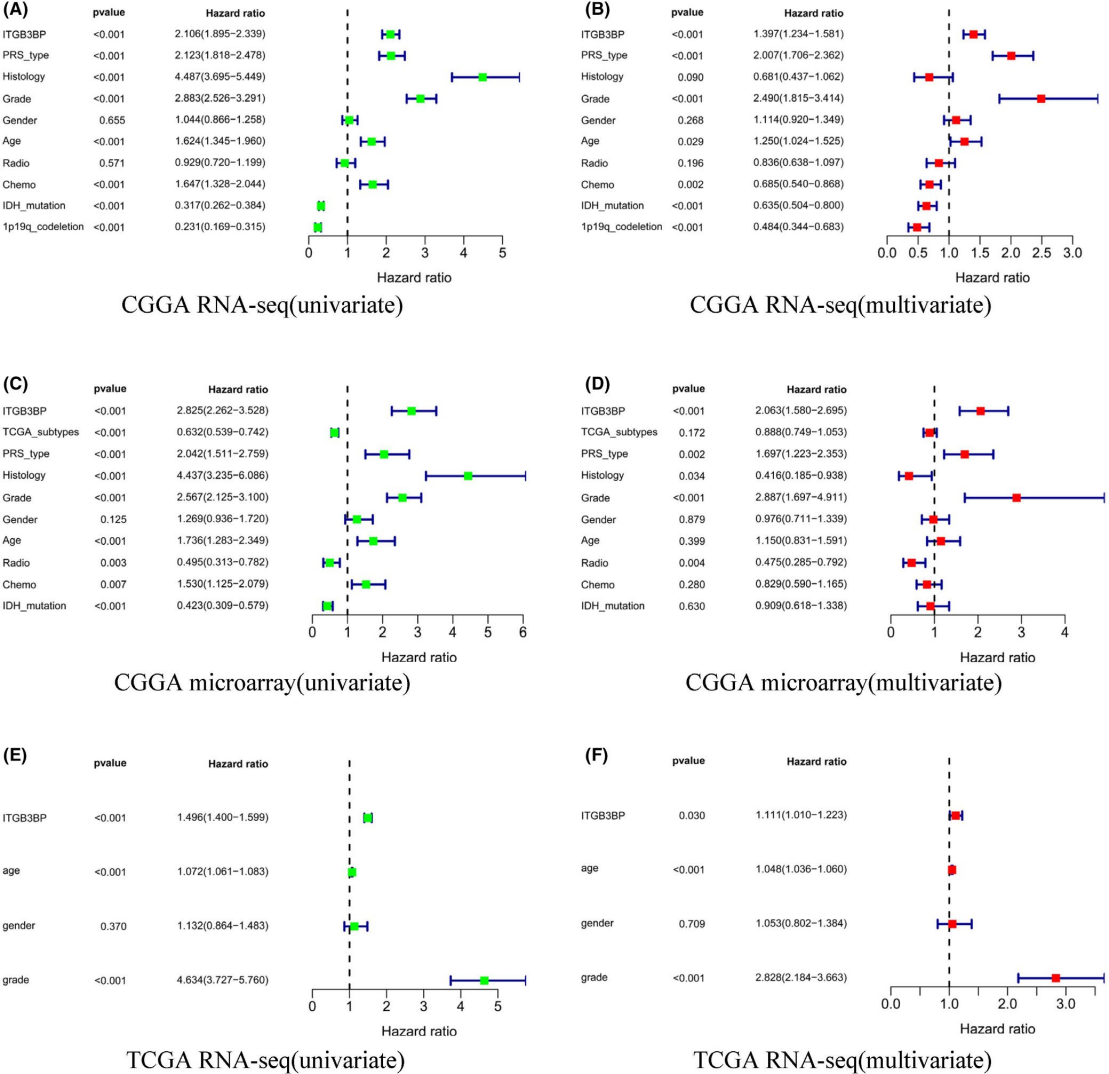
*ITGB3BP* up‐regulation was an independent risk factor for patients with glioma. A, C and E are univariate analysis results, and B, D and F are multivariate analysis results based on three different databases (CGGA RNA‐Seq; CGGA RNA‐microarray; TCGA RNA‐Seq). CGGA, Chinese Glioma Genome Atlas; *ITGB3BP*, integrin subunit β3‐binding protein; TCGA, The Cancer Genome Atlas

To confirm the effect of high expression of *ITGB3BP* on the prognosis of patients, we obtained three datasets from the CGGA and TCGA databases and divided them into two groups according to the expression level of *ITGB3BP*. Kaplan‐Meier survival curves were drawn to identify the OS of each group, as shown in Figure 3A–C. We found that the OS of the up‐regulated group was lower than that of the down‐regulated group (*p* < 0.001). This indicates that the up‐regulation of *ITGB3BP* is related to poor outcome. Subsequently, ROC curves were plotted to determine whether the expression level of *ITGB3BP* has clinical diagnostic value for glioma. As shown in Figure 3D–F, *ITGB3BP* was up‐regulated and had a strong predictive value in the 3‐ and 5‐year outcomes of glioma in the CGGA‐Seq (AUC > 0.7), CGGA microarray (AUC > 0.8) and TCGA‐Seq datasets (AUC > 0.8). The above results indicate that the up‐regulation of *ITGB3BP* has high prognostic value. Moreover, to verify whether *ITGB3BP* expression is an independent factor affecting the survival of glioma patients, we conducted univariate and multivariate analyses based on different datasets, and the results showed that *ITGB3BP* up‐regulation could be an independent risk factor for the prognosis of glioma (Figure 4A–F). Hence, the above results show that *ITGB3BP* is an oncogene associated with poor prognosis in glioma patients.

### 3.4 GSEA revealed the effect of high expression of *ITGB3BP* on the cell signalling pathway

**FIGURE 5:**
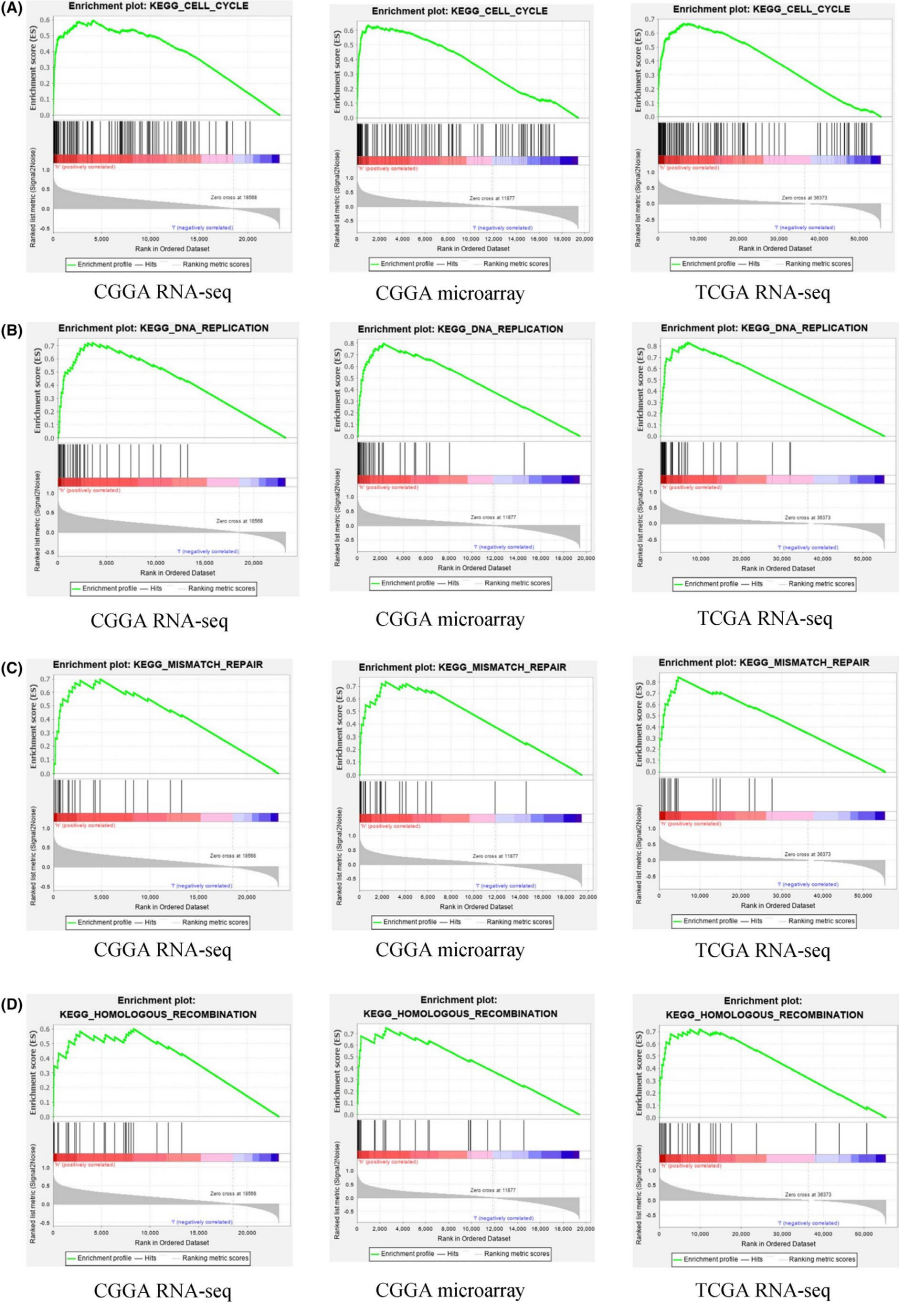
Gene Set Enrichment Analysis enrichment plots. A: cell cycle. B: DNA replication. C: mismatch repair. D: homologous recombination

Based on the above results, we clarified that the overexpression of *ITGB3BP* can lead to poor prognosis in glioma patients; however, the mechanism by which *ITGB3BP* participates in regulating the pathological progress of glioma needs to be further explained. GSEA is one of the most common bioinformatics methods that can provide the biological regulatory function of target genes. Therefore, we used GSEA to evaluate three different datasets from the CGGA and TCGA databases to reveal the effect of *ITGB3BP* on cell signalling pathways. GSEA showed that the cell cycle, DNA replication, mismatch repair and homologous recombination pathways were activated in the *ITGB3BP* high expression group. Activation of these four cellular signalling pathways can promote the malignant progression of tumour cells. The results presented by the three different databases had remarkable consistency (NES > 1.5 and *p* < 0.05), which makes the objectivity of the data verified by each other (Figure 5 and Table 1).

**TABLE 1:** Cell signalling pathways that *ITGB3BP* be enriched

Gene set name	CGGA RNA‐seq		CGGA microarray		TCGA RNA‐seq	
	NES	NOM *p*‐val	NES	NOM *p*‐val	NES	NOM *p*‐val
Cell‐cycle	1.792	0.01212	1.913	0.00610	1.992	0.00204
DNA‐Replication	1.802	0.01183	1.797	0.00205	1.881	0.00576
Mismatch‐repair	1.733	0.01018	1.718	0.01210	1.949	0
Homologous‐recombination	1.593	0.03259	1.912	0	1.760	0.01004

Note

Gene sets with NOM *p* value <0.05 was considered as significantly enriched.

Abbreviations: NES, normalized enrichment score; NOM, nominal.

### 3.5 Functional correlation of *ITGB3BP* and other genes with potential therapeutic compounds

**FIGURE 6:**
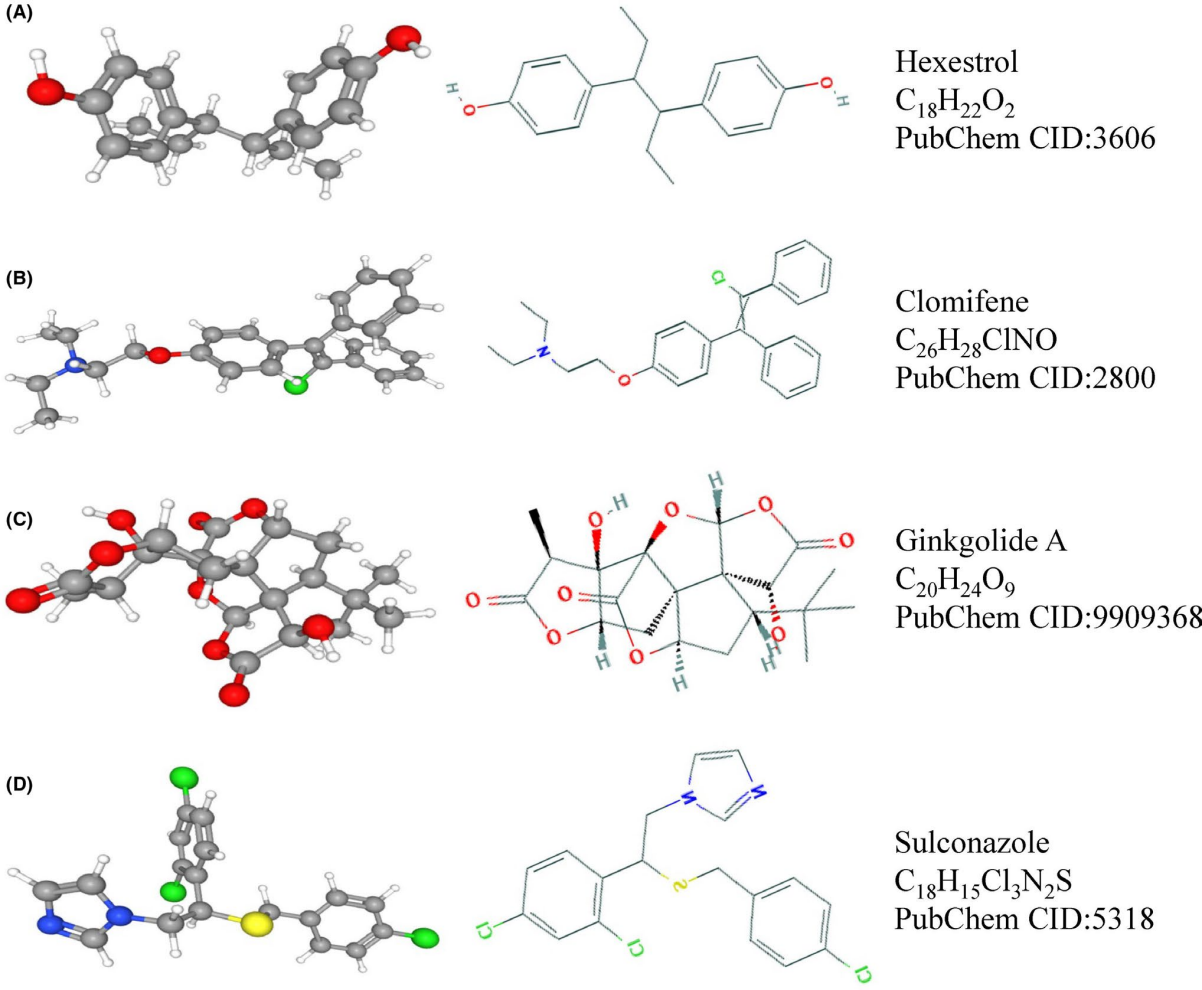
Four small‐molecule drugs with potential therapeutic effects on glioma. A: Hexestrol. B: Clomifene. C: Ginkgolide A. D: Sulconazole

To further explore the functions of *ITGB3BP*, we investigated genes co‐expressed with *ITGB3BP* using correlation analysis. As shown in Figure S2 and Table 2, the expression of *ITGB3BP* was positively correlated with the expression of *GNG5*, *RBBP8*, *AK2* and *HMGB2*, but negatively correlated with the expression of *HIST3H2BB*, *FBXW4* and *TMEM56*. Based on the results of the co‐expression analysis and online analysis via CMap, we identified four small‐molecule compounds with potential effects on *ITGB3BP* expression (Table 3), and their molecular and structural formulas were identified at https://pubchem.ncbi.nlm.nih.gov/ (Figure 6). These compounds are expected to become new treatments for glioma in the future.

**TABLE 2:** Co‐expressed genes with *ITGB3BP* in glioma

Gene	Cor	*p* value
HSPB11	0.823	9.04E−252
PCNA	0.821	5.10E−249
GNG5	0.821	5.04E−249
SNRNP40	0.819	1.62E−247
RPF1	0.818	4.23E−246
YTHDF2	0.816	2.99E−244
MGME1	0.815	8.00E−243
HMGB2	0.813	2.15E−240
AK2	0.813	1.06E−240
RBBP8	0.812	8.60E−240
RIMS1	−0.547	1.89E−80
CDYL2	−0.529	1.55E−74
TUB	−0.475	2.15E−58
RN7SL3	−0.474	3.50E−58
ATRNL1	−0.469	1.07E−56
RN7SL4P	−0.463	4.23E−55
CDR1	−0.45	6.43E−52
TMEM56	−0.441	1.02E−49
FBXW4	−0.433	7.93E−48
HIST3H2BB	−0.432	1.58E−47

Note

Cor >0 was positively correlated and cor <0 was negatively correlated.

**TABLE 3:** Four small‐molecule compounds were identified as potential drugs for gliomas treatment in CMap analysis

Cmap name	Enrichment	*p* value
Hexestrol	−0.828	0.00165
Clomifene	−0.756	0.00716
Ginkgolide A	−0.763	0.00649
Sulconazole	−0.867	0.00062

Abbreviation: CMap, connectivity map.

### 3.6 Meta‐analysis verified the prognostic risk of *ITGB3BP* in patients with glioma

**FIGURE 7:**
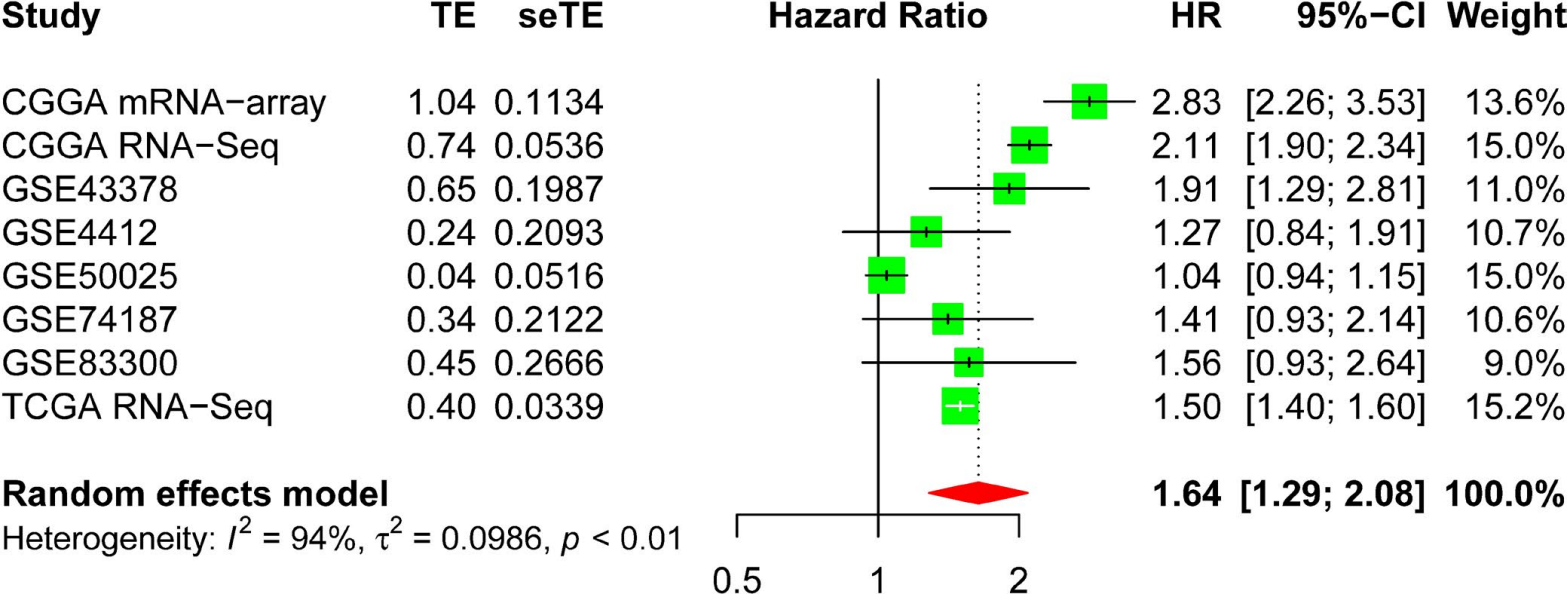
Forest plot of association between high *ITGB3BP* expression and poor prognosis in glioma patients based on the CGGA RNA‐Seq, CGGA microarray, TCGA RNA‐Seq and GEO datasets. CGGA, Chinese Glioma Genome Atlas; GEO, Gene Expression Omnibus; *ITGB3BP*, integrin subunit β3‐binding protein; TCGA, The Cancer Genome Atlas

To clarify the impact of *ITGB3BP* on the survival prognosis of patients with glioma, we further used a meta‐analysis based on publicly available data for verification. However, since no previous studies have reported the relationship between high *ITGB3BP* expression and survival time in glioma patients, we could not incorporate any published data into the meta‐analysis. Therefore, we queried the original data for survival time and survival status from the public databases and incorporated them into the meta‐analysis to obtain more evidence to support our research results that *ITGB3BP* is a risk factor for glioma patients. Finally, eight different datasets (GSE43378: 50 patients; GSE74187: 60 patients; GSE4412: 85 patients; GSE50025: 34 patients; GSE83300: 50 patients; CGGA RNA‐Seq: 748 patients; CGGA microarray: 268 patients; and TCGA RNA‐Seq: 653 patients) were obtained, and 1948 glioma tissue samples were included in the meta‐analysis. As shown in Figure 7, the pooled HR and 95% CI of *ITGB3BP* overexpression and OS were 1.64 (1.29–2.08) in 1948 patients with glioma. Hence, we believe that the high expression of *ITGB3BP* is a powerful predictor of good survival and prognosis in patients with glioma.

### 3.7 Relationship of *ITGB3BP* with immune cells

**FIGURE 8:**
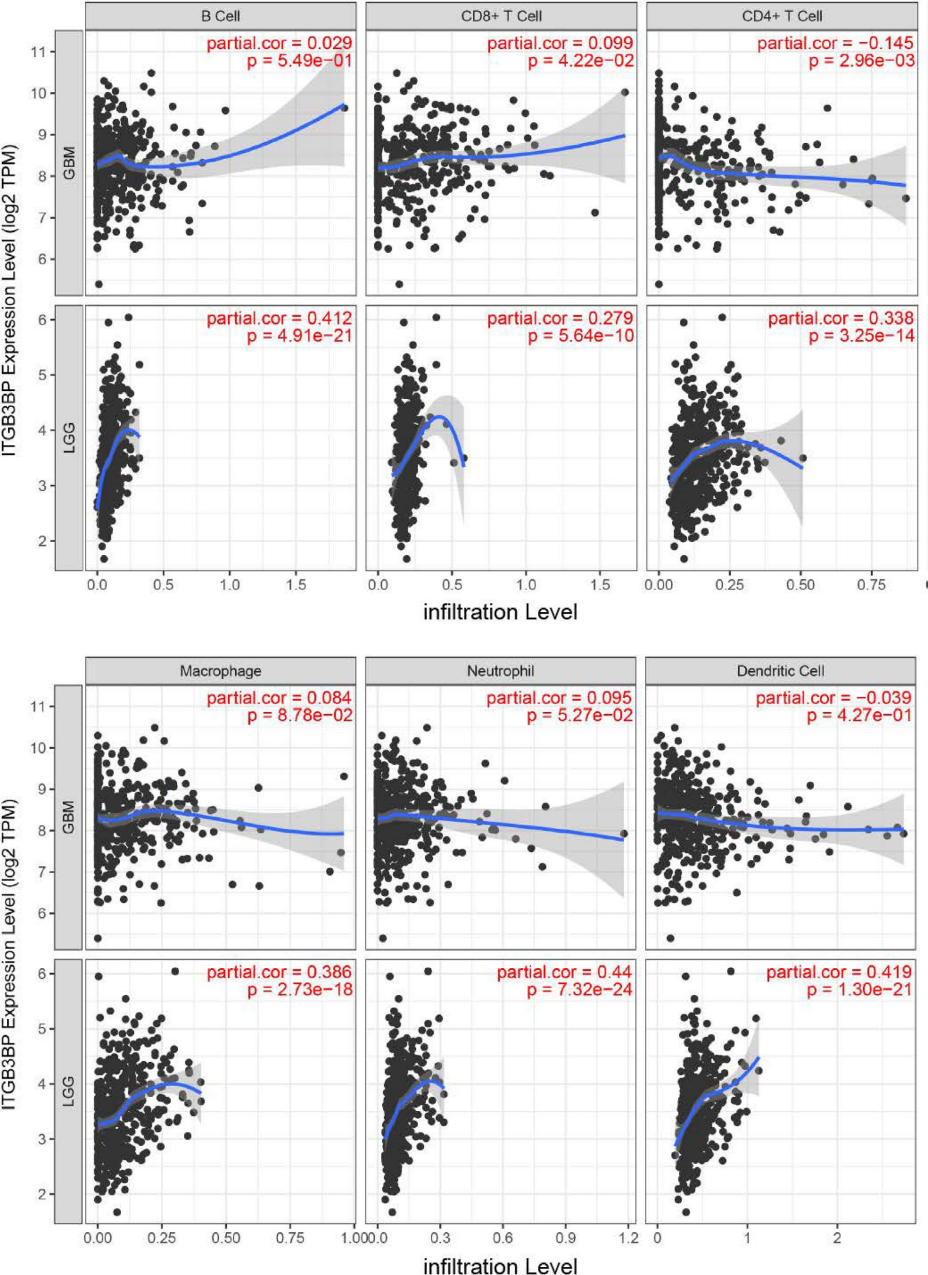
Expression of *ITGB3BP* was related to immune cell infiltration

We explored the correlation between *ITGB3BP* expression levels and immune cell infiltration using the TIMER database. The results showed that *ITGB3BP* expression was positively correlated with CD8^+^ T cell infiltration and negatively correlated with CD4^+^ T cell infiltration in GBM. In LGG, *ITGB3BP* expression was positively associated with the infiltration of B cells, CD8^+^ T cells, neutrophils, CD4^+^ T cells, macrophages and dendritic cells (Figure 8). In addition, we explored the relationship between *ITGB3BP* and well‐known immune checkpoints. The expression of *ITGB3BP* was positively correlated with the expression of *CD274*, *PDCD1* and *PDCD1LG2* in LGG and negatively correlated with the expression of *CD274* in GBM (Figure S3). These results suggest that the effects of *ITGB3BP* on immune cells and immune checkpoints vary among different grades of glioma, possibly due to the considerable heterogeneity between GBM and LGG; however, a detailed mechanism needs to be further verified.

## 4 DISCUSSION

The main characteristics of tumours are increased proliferation, angiogenesis, migration and invasion.^31^ Presently, studies have confirmed that *ITGB3BP* is related to the pathogenesis of various tumours^19^, ^20^, ^21^, ^22^; however, the relationship between *ITGB3BP* and glioma has not been reported. Therefore, this study aimed to analyse the clinical and prognostic effects of *ITGB3BP* on glioma. For the first time, we revealed a high expression level of *ITGB3BP* in glioma from the GEPIA, GEO and HPA databases. Further, we confirmed by RT‐qPCR that *ITGB3BP* expression was significantly increased in glioma cells and tissues. The above evidence demonstrated that *ITGB3BP* was overexpressed in glioma. Second, our analysis results showed that the expression level of *ITGB3BP* was closely related to a series of important clinical features of glioma, and survival analysis showed that increased expression of *ITGB3BP* was not conducive to the survival of patients. Meta‐analysis results also further confirmed that abnormally high expression of *ITGB3BP* can lead to a decline in the survival rate of patients with glioma. Hence, we believe that the overexpression of *ITGB3BP* plays a critical role in the poor prognosis of glioma patients. Consistently, through univariate, multivariate and ROC analyses, we concluded that *ITGB3BP* could be used as an independent predictive factor for the prognosis of glioma, with a specific diagnostic value. Finally, we used GSEA and TIMER database analysis to explore the possible mechanism of *ITGB3BP* in glioma and identified potential therapeutic compounds that may target *ITGB3BP* by CMap analysis. This is the first study to reveal the relationship between *ITGB3BP* and glioma, to the best of our knowledge. *ITGB3BP* may be an oncogene that affects the pathological process of glioma, but the specific mechanism still needs to be further explored.

Prior studies have noted that *ITGB3BP* affects the occurrence and development of tumours through different signal transduction pathways. For example, Yamanoi et al. found that the abnormally high expression of *ITGB3BP* could activate the hedgehog pathway in ovarian cancer, which led to the resistance of ovarian cancer cells to chemoradiotherapy.^32^ In this study, we confirmed that *ITGB3BP* could lead to a poor prognosis of glioma, but the regulatory effect of *ITGB3BP* overexpression on the cell signalling pathway needs to be further elucidated. The results of GSEA suggested that the high *ITGB3BP* expression group exhibited increased activity of a variety of cellular signalling pathways related to malignant tumour progression. For instance, the cell cycle pathway plays a vital role in the proliferation and apoptosis of tumour cells.^33^ Previous studies have shown that cedrelone can cause glioma cell cycle arrest and effectively inhibit the cell cycle pathway.^34^, ^35^ DNA replication can be divided into three main steps: initiation, extension and termination. The interruption of DNA replication can lead to genome instability, leading to many congenital diseases and cancer.^36^ Numerous experimental and clinical studies have shown that most tumours experience abnormal DNA replication.^36^ In addition, mismatch repair is the most important method of DNA repair and is widely observed in tumours.^37^, ^38^ Resistance to TMZ is regulated by DNA mismatch repair in glioma. Moreover, research has shown that homologous recombination plays an essential role in ovarian cancer, fallopian tube cancer and peritoneal cancer.^39^ Collectively, we discovered multiple tumour‐related signalling pathways via GSEA, which suggests that *ITGB3BP* may be involved in the tumourigenesis of glioma through these pathways.

Glioma involves an extremely complicated pathological process, and a variety of genes play a significant role in its pathogenesis. In the gene co‐expression analysis, we found many genes co‐expressed with *ITGB3BP* in glioma, which play a potential role in malignant progression. Among the positively correlated genes, evidence shows that *GNG5*, *HMGB2*, *PCNA* and *HSPB11* can induce the proliferation and metastasis of glioma, causing poor prognosis and reducing the OS of patients with glioma.^40^, ^41^, ^42^ In addition, among the negatively correlated genes, *CDR1* decreases the proliferation, migration and invasion of glioma cells and promotes cell apoptosis.^43^ Based on the above studies, we further confirmed that *ITGB3BP*, as an oncogene, plays a critical role in the malignant process of glioma, together with the co‐expressed oncogenes. Moreover, these results further reveal the potential clinical value of drugs targeting *ITGB3BP*.

To explore the clinical value of *ITGB3BP*, using the CMap online drug analysis tool, we identified four small‐molecule drugs that may inhibit *ITGB3BP* expression: hexestrol, clomifene, ginkgolide A and sulconazole. The anti‐tumour effects of these drugs have been confirmed in previous studies. First, hexestrol can be used as a potential anti‐carcinogen in lymphocytic leukaemia.^44^ Second, Zheng et al. suggested that clomifene could be used as an inhibitor of mutant isocitrate dehydrogenase to inhibit the growth of tumours, such as glioma, acute myeloid leukaemia and colorectal cancer.^45^ Third, ginkgolide A has a significant anti‐proliferative effect on serous ovarian cancer cells.^46^ Finally, sulconazole inhibits the proliferation of breast cancer stem cells.^47^ Therefore, these drugs may have therapeutic effects on glioma.

Although we have no direct evidence to confirm this hypothesis, the CMap online analysis method can quickly reveal strong correlations between drugs and diseases by comparing gene expression profiles, which is conducive to developing new drugs. With an in‐depth understanding of drugs and diseases, some old drugs have been used for new applications in recent years. Metformin, for example, controls blood glucose and can now be used as an adjunct treatment for breast cancer and colorectal cancer.^48^ In addition, aspirin, a traditional anti‐inflammatory and anti‐platelet drug, assists in the prognosis of colorectal cancer.^49^ Therefore, we believe that the above four small‐molecule drugs have potential therapeutic effects for glioma patients via inhibition of *ITGB3BP* expression.

Immunosuppressive cells infiltrating the TME are key factors in the formation of the tumour‐suppressive immune microenvironment. These immunosuppressive cells include CD4^+^ T cells, CD8^+^ T cells, B cells, neutrophils, dendritic cells and macrophages, which can assist immune evasion, thereby promoting the occurrence and development of tumours.^50^, ^51^ Previous studies have pointed out that tumour‐infiltrating immune cells are key regulators of tumour growth and progression, which are related to the biological behaviour of glioma and patient survival.^52^ In this study, we observed that *ITGB3BP* expression levels were positively correlated with CD8^+^ T cell infiltration (GBM: *r* = 0.099, *p* = 0.0422; LGG: *r* = 0.279, *p* < 0.01). Recent studies have reported that activated CD8^+^ T cells can recruit microglia to secrete CCL5 by secreting CCL4, thereby playing a key role in the survival of LGG stem cells. However, the effect of *ITGB3BP* on the immune microenvironment in GBM is relatively limited compared with that in LGG, probably because of the large amount of heterogeneity between GBM and LGG, although both are glioma subtypes. Collectively, *ITGB3BP* has a regulatory effect on the formation of the LGG immune microenvironment. Whether *ITGB3BP* can participate in the regulation of LGG immune escape remains to be confirmed.

Although our current results have increased our understanding of the relationship between *ITGB3BP* and glioma, this study still had some limitations. First, to fully understand the specific mechanism of action of *ITGB3BP* in the development and progression of glioma, we need to consider various clinical factors, such as the specific treatment of patients. Since the data came from different research centres, it was difficult to overcome the lack of clinical sample information in the public databases. Second, although the mutual verification of multiple public databases can compensate for the inherent shortcomings of single‐centre research, there are still some shortcomings, especially the inconsistency of intervention measures and the lack of clinical information. However, the current results are encouraging and deserve attention in research efforts to identify promising prognostic biomarkers for glioma.

## 5 CONCLUSION

Our study revealed that the overexpression of *ITGB3BP* was associated with the molecular and clinical characteristics of malignancy and predicted poor prognosis in glioma. Collectively, these findings provide new insight into the role in glioma of *ITGB3BP*, which might serve as a potential biomarker and novel therapeutic target for diagnosis and treatment.

## CONFLICT OF INTEREST

The authors declare that they have no competing interests.

## AUTHOR CONTRIBUTIONS

**Zhendong Liu:** Software (equal); Writing – review & editing (equal). **Binfeng Liu:** Data curation (equal); Writing – original draft (equal). **Lu Bian:** Data curation (equal); Writing – original draft (equal). **Hongbo Wang:** Software (equal); Validation (equal). **Yulong Jia:** Writing – review & editing (equal). **Yubo Wang:** Writing – review & editing (equal). **Wang Zhang:** Formal analysis (equal). **Yanbiao Wang:** Formal analysis (equal). **Zhibin Han:** Validation (equal). **Xingbo Cheng:** Validation (equal). **Xiaoyu Lian:** Visualization (equal). **Zhishuai Ren:** Visualization (equal). **Yanzheng Gao:** Funding acquisition (equal); Supervision (equal).

## Supporting information

Figure S1

Figure S2

Figure S3

Table S1

Table S2

Table S3

## Data Availability

The datasets supporting the conclusions of this article are available in the TCGA database [http://www.cgga.org.cn/], CGGA database [http://www.cgga.org.cn/] and GEO [https://www.ncbi.nlm.nih.gov/geo/] or from the corresponding author on reasonable request.
