# Supplementary figures and images for: ITGB3BP is a potential biomarker associated with poor prognosis of glioma
Source: J Cell Mol Med. 2021 Dec 24;26(3):813–27. doi: 10.1111/jcmm.17127 (PMC8817129; doi:10.1111/jcmm.17127)

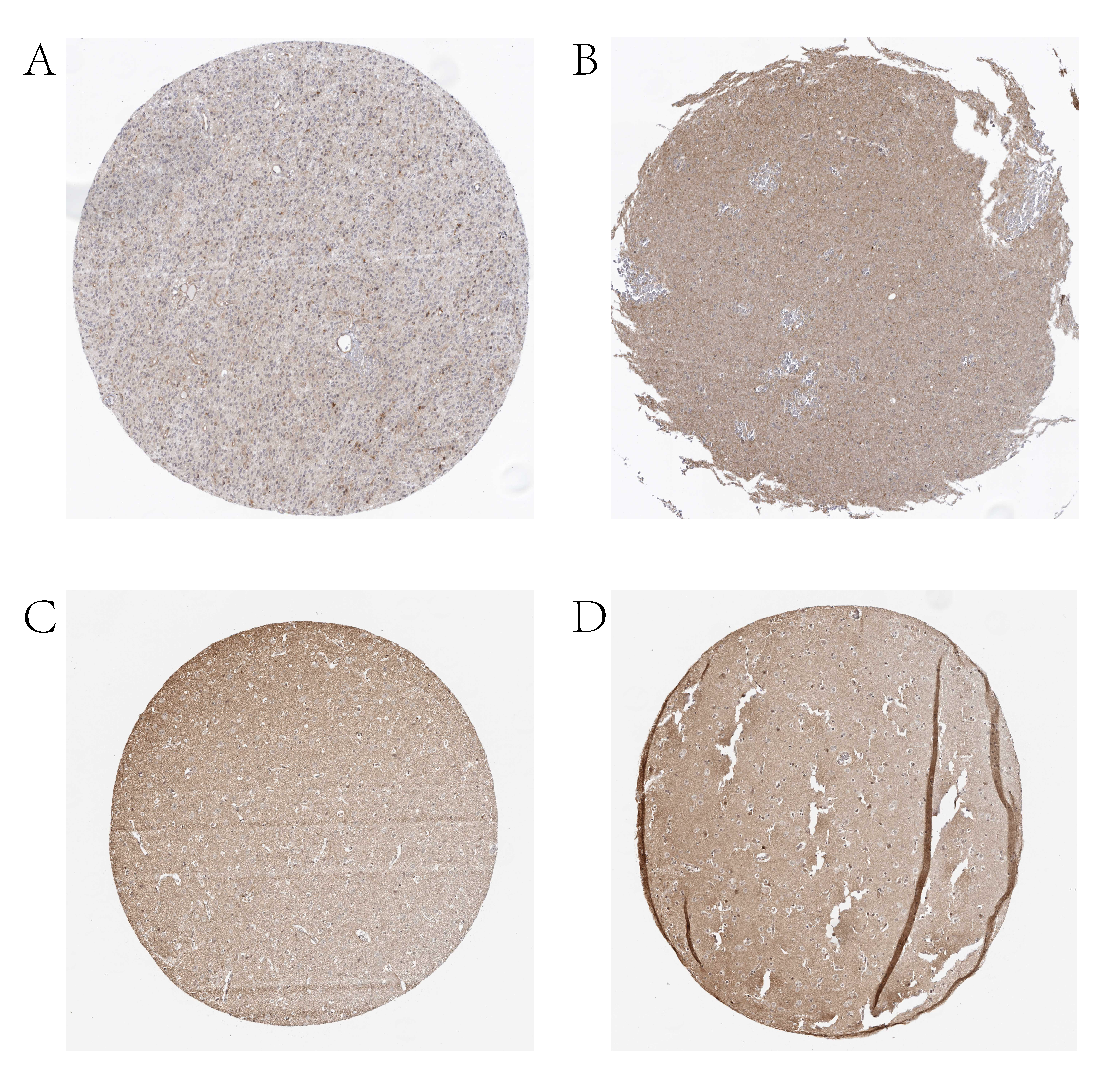

Supplement: Supplementary file 1 — Figure S1 [file JCMM-26-813-s001.tif]

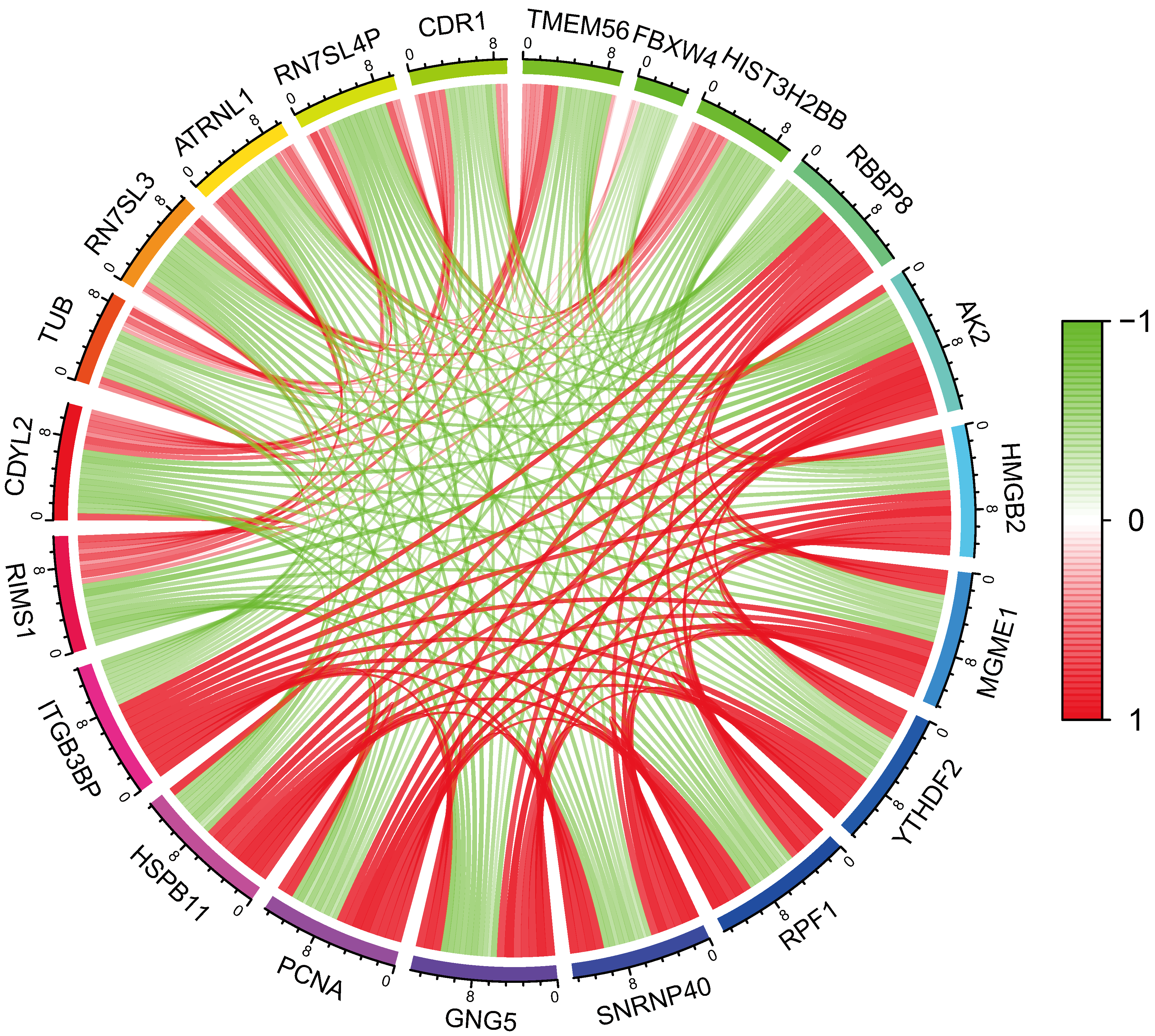

Supplement: Supplementary file 2 — Figure S2 [file JCMM-26-813-s004.tif]

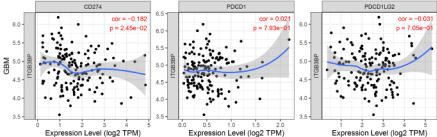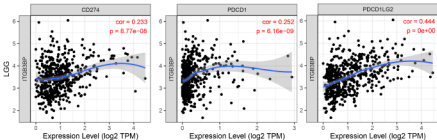

Supplement: Supplementary file 3 — Figure S3 [file JCMM-26-813-s005.pdf]
