# Supplementary material for: ITGB3BP is a potential biomarker associated with poor prognosis of glioma
Source: J Cell Mol Med. 2021 Dec 24;26(3):813–27. doi: 10.1111/jcmm.17127 (PMC8817129; doi:10.1111/jcmm.17127)
Supplement: Supplementary file 4 — Table S1 [file JCMM-26-813-s002.docx]

Table S1. Characteristics of patients with glioma based on CGGA

| Characteristics |  | Number of cases | Percentages (%) |
| --- | --- | --- | --- |
| Gender | Male | 306 | 40.91 |
|  | Female | 442 | 59.09 |
| Age | <=41 | 341 | 45.59 |
|  | >41 | 407 | 54.41 |
| Grade | WHO II | 218 | 29.14 |
|  | WHO III | 240 | 32.09 |
|  | WHO IV | 290 | 38.77 |
| PRS_type | Primary | 501 | 66.98 |
|  | Recurrent | 222 | 29.68 |
|  | Secondary | 25 | 3.34 |
| Radio_status | Yes | 625 | 83.56 |
|  | No | 123 | 16.44 |
| Chemo_status | Yes | 520 | 69.52 |
|  | No | 228 | 30.48 |
| Histology | Astrocytoma | 55 | 7.35 |
|  | Anaplastic astrocytoma | 39 | 5.21 |
|  | Anaplastic Oligodendroglioma | 22 | 2.94 |
|  | Anaplastic oligoastrocytoma | 80 | 10.70 |
|  | Glioblastoma | 175 | 23.40 |
|  | Oligodendroglioma | 35 | 4.68 |
|  | oligoastrocytoma | 95 | 12.70 |
|  | relapse astrocytoma | 20 | 2.67 |
|  | relapse Anaplastic astrocytoma | 36 | 4.81 |
|  | relapse Anaplastic Oligodendroglioma | 15 | 2.01 |
|  | relapse Anaplastic oligoastrocytoma | 48 | 6.42 |
|  | relapse Oligodendroglioma | 90 | 12.03 |
|  | relapse Oligodendroglioma | 4 | 0.53 |
|  | relapse oligoastrocytoma | 9 | 1.20 |
|  | Secondary relapse Oligodendroglioma | 25 | 3.34 |
| IDH_mutation_status | Mutant | 409 | 54.68 |
|  | Wildtype | 339 | 45.32 |
| 1p19q_codeletion_status | Codel | 155 | 20.72 |
|  | Non-codel | 593 | 79.28 |
