# Supplementary material for: ITGB3BP is a potential biomarker associated with poor prognosis of glioma
Source: J Cell Mol Med. 2021 Dec 24;26(3):813–27. doi: 10.1111/jcmm.17127 (PMC8817129; doi:10.1111/jcmm.17127)
Supplement: Supplementary file 6 — Table S3 [file JCMM-26-813-s006.docx]

Table S3. Characteristics of patients with glioma based on TCGA

| Characteristics |  | Number of cases | Percentages (%) |
| --- | --- | --- | --- |
| Gender | Male | 377 | 57.73 |
|  | Female | 276 | 42.27 |
| Age | <=51 | 394 | 60.34 |
|  | >51 | 259 | 39.66 |
| Grade | WHO II | 238 | 36.45 |
|  | WHO III | 256 | 39.20 |
|  | WHO IV | 159 | 24.35 |
